# Microbial Community Assembly Mechanisms of Groundwater Under Salinity–Oxygen Stress in the Golmud River Watershed, Northwest China

**DOI:** 10.3390/life15081301

**Published:** 2025-08-15

**Authors:** Liang Guo, Haisong Fang, Yuanyuan Ding, Chunxue An, Nuan Yang

**Affiliations:** College of Geology and Environment, Xi’an University of Science and Technology, Xi’an 710054, China; guoliang@xust.edu.cn (L.G.);

**Keywords:** microbial community, salinity–oxygen stress, ecological process, hydrochemical characteristic, Golmud River watershed

## Abstract

The mechanisms underlying groundwater microbial community assembly have long attracted attention in earth, environmental, and ecological studies. Nevertheless, limited knowledge is available regarding microbial community assembly within the intact groundwater flow systems in arid regions. In this study, long-term hydrochemical data and microbial community profiles were integrated to unravel the assembly processes and driving forces mediating microbial communities in the Golmud River watershed. Our results indicated that hydrochemical conditions gradually transitioned from oxidizing to reducing environments along the groundwater flow path, as evidenced by a 28.57% and 65.45% decrease in DO and ORP, respectively. Major ions, represented by TDS, displayed minimal variations in phreatic (519.72 ± 16.83 mg/L) and artesian groundwater (486.01 ± 27.71 mg/L), followed by pronounced enrichment in high-salinity groundwater (TDS: 316,112.74 ± 12,452.19 mg/L). *Gammaproteobacteria* and *Actinobacteria* declined markedly from phreatic (51.69 ± 6.83% and 9.54 ± 3.40%, respectively) to high-salinity groundwater (13.97 ± 3.70% and 4.77 ± 2.46%). Conversely, halophiles such as *Halobacteria* and *Parcubacteria* were rarely detected in low-TDS groundwater, but increased sharply in high-salinity groundwater, reaching 23.22 ± 10.42% and 8.34 ± 3.71%, respectively. Deterministic processes primarily controlled groundwater microbial communities across hydrochemical conditions (relative importance > 50%, NST index < 50%). Microbial co-occurrence networks revealed increasingly tight interactions and intensified competition among communities, driven by accumulated salinity–oxygen stress along the groundwater flow path. This study emphasizes the role of deterministic processes in shaping groundwater microbial community structure, particularly the impact of salinity–oxygen stress. Our findings advance the current understanding of the mechanisms by which hydrochemical processes shape groundwater microbial assemblages.

## 1. Introduction

The subsurface environment hosts a vast number of prokaryotes, accounting for approximately 2% to 19% of Earth’s total biomass [1]. Functioning as key regulators of the subsurface ecosystem, microorganisms actively participate in subsurface biogeochemical cycling through electron transfer driven by mineral–microbial interactions and metabolic modulation of elemental processes, thereby exerting significant influence on global elemental cycles [2,3]. Niche-based theory emphasizes the dominant role of deterministic processes, where microbial community is jointly shaped by species traits, interspecific interactions (e.g., competition, predation, symbiosis, and ecological trade-offs), and environmental factors such as pH, temperature, salinity, and moisture [4]. In contrast, neutral theory posits that community assembly is primarily governed by stochastic processes that are independent of species traits, including birth, death, migration, extinction, and speciation [5]. Although it is widely acknowledged that both deterministic and stochastic processes contribute to microbial assembly, their relative importance in shaping microbial community structure and function remains under debate [6,7]. Understanding microbial community assembly mechanisms in the subsurface is one of the key goals for a wide range of earth, environmental, and ecological studies [8].

The assembly of microbial communities in subsurface aquifers is widely recognized as being shaped by the interplay between deterministic and stochastic processes, with their relative contributions strongly influenced by habitat characteristics. Microbial communities across the vadose, fluctuation, and saturated zones exhibited a habitat-dependent assembly mechanism [9]. Deterministic processes dominated in the vadose zone, while stochastic processes prevailed in the fluctuation and saturated zones. Environmental gradients such as stress intensity and habitat heterogeneity can significantly modulate the relative contributions of deterministic and stochastic processes, thereby shaping the overall framework of community assembly. For instance, stochastic processes were identified as the primary drivers of community structure in a typical groundwater system in Oak Ridge, Tennessee, USA, accounting for over 60% on average; however, their influence decreased with increasing environmental stress (from 60% to 41%), accompanied by a substantial increase in the contribution of deterministic processes (from 16.5% to 66.9%) [10]. Multiple studies have indicated that key environmental factors, such as total dissolved solids (TDS) and pH, are often the primary environmental drivers shaping variations in groundwater microbial community structure [10,11]. However, current research remains limited in understanding microbial community assembly within complete groundwater flow systems of Quaternary aquifers in arid regions. These groundwater systems, largely isolated from surface disturbances, offer ideal conditions for disentangling community assembly mechanisms and assessing the relative contributions of deterministic and stochastic processes. Addressing this knowledge gap is critical for determining whether deterministic or stochastic processes play the dominant role in shaping groundwater microbial communities.

Our previous research revealed clear spatial patterns in microbial community composition along the groundwater flow path within the porous Quaternary aquifer of Golmud, situated in the largest arid basin on the Tibetan Plateau. These patterns were primarily driven by hydrochemical variables such as dissolved oxygen, redox potential, pH, and salinity [12]. However, the extent to which deterministic and stochastic processes govern the spatial distribution patterns of microbial communities along the groundwater flow path at the regional scale remains poorly understood. To address this issue, we collected and analyzed multi-batch datasets of groundwater chemistry and microbial communities from phreatic, artesian, and high-salinity groundwater samples spanning 2015–2023. By applying multivariate statistical approaches and community assembly theory, this study aims to uncover the dominant ecological processes and environmental drivers shaping microbial community assembly in aquifers with high environmental heterogeneity. The objectives of this study are as follows: (1) to integrate multi-batch datasets of hydrochemical variables and microbial communities and analyze their spatial distribution patterns, and (2) to identify the key ecological processes driving community assembly and their associations with environmental gradients. This study will enhance our understanding of the dominant ecological mechanisms governing microbial community assembly along Quaternary groundwater flow systems in arid regions, thereby providing new insights into microorganism–environment interactions in hydrologically connected subsurface ecosystems.

## 2. Materials and Methods

### 2.1. Study Area and Sample Collection

**Figure 1:**
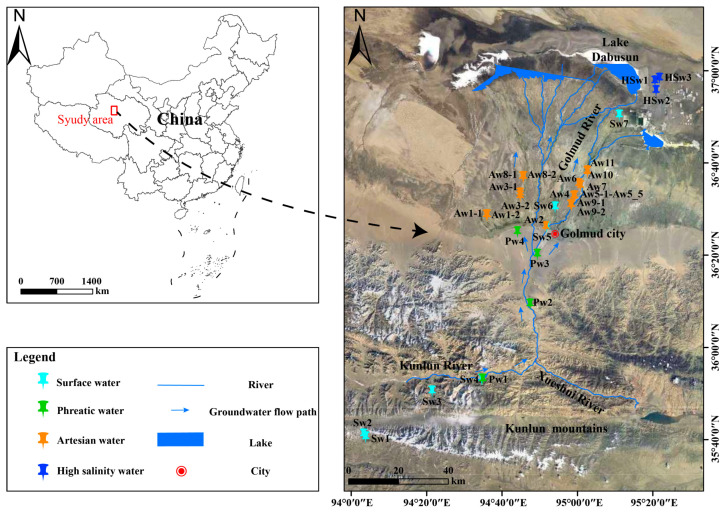
Geological and topographic map of the study area with sampling locations along the groundwater flow path.

The Qaidam Basin is a large, enclosed inland basin located on the northeastern margin of the Tibetan Plateau and bounded by the Qilian Mountains to the north, the Kunlun Mountains to the south, and the Altun Mountains to the west [13]. Due to the barrier of the surrounding mountains, the transport of moisture from the Pacific, Indian, and Atlantic Oceans is largely obstructed. The Qaidam Basin experiences extremely low precipitation, with an average annual rainfall of only 42.3 mm under the typical plateau continental climate, while the potential evaporation is reaching a remarkable 2626.9 mm [12,14,15]. This research selected the Golmud River watershed, the second-largest river in the Qaidam Basin, as the study area (Figure 1). The Golmud River watershed is located in the southern part of the basin and originates from the Kunlun Mountains. Originating in the mountainous area, the river flows through the piedmont alluvial fan and the alluvial–lacustrine plain in sequence and ultimately discharges into Dabusun Lake [14]. The direction of groundwater flow in the basin generally aligns with the surface runoff [12]. The groundwater aquifer system exhibits two typical structural types: a thick unconfined aquifer composed of gravel and sand layers in the alluvial fan, and a multi-layered confined aquifer composed of clay, sand, and gravel layers in the alluvial–lacustrine plain [12,14]. Glaciers and snowmelt from the Kunlun Mountains serve as the primary recharge sources for groundwater in the Golmud River watershed.

Field investigations of the watershed-scale groundwater system were conducted in the summer seasons of 2015, 2016, 2022, and 2023, during which groundwater samples were collected for both microbial and hydrochemical analyses along a generalized groundwater flow path (Figure 1). In this study, a total of 26 groundwater sampling sites were established, including 4 phreatic groundwater, 19 artesian groundwater, and 3 high-salinity groundwater sites. Additionally, 7 surface river waters were included for comparison. Groundwater in confined aquifers discharges as artesian well flows. Detailed information on the sampling locations and corresponding sampling years is provided in Table S1. It is worth noting that site Sw7, located near Dabusun Lake, was excluded from the hydrochemical dataset due to its high salinity, which could distort the overall chemical characteristics of river water. However, the microbial community at this site exhibited features typical of high-salinity groundwater, and thus it was classified as “high-salinity water” in the bioinformatics analysis. For the classification of groundwater salinity, this study adopts the widely recognized hydrochemical standard, explicitly defining “high-salinity groundwater” as groundwater with total dissolved solids (TDS) ≥ 10,000 mg/L, which falls into the saline and brine categories. This threshold can effectively distinguish extreme salinity levels significantly higher than freshwater and provide a clear basis for interpreting the ecological impacts of high-salinity conditions in the study area. For molecular biological analysis, 2–3 L of groundwater was filtered on-site through pre-sterilized membrane filters (0.22 μm) to collect microbial biomass. The membrane was sealed and stored on dry ice and processed for DNA extraction in the laboratory within 72 h, following protocols previously described [16]. Water samples for hydrochemical analysis were filtered using 0.45 μm membrane filters and subdivided into two aliquots: 500 mL for cation analysis (acidified with analytical-grade HNO_3_ to prevent oxidation and precipitation), and 500 mL for anion analysis. Field blanks were acidified in the same manner to minimize the risk of acid contamination. All hydrochemical samples were stored in non-headspace polyethylene bottles and sealed with parafilm to prevent air exposure [14].

### 2.2. Geochemical Parameters Analysis

Hydrochemical parameters were determined through both in situ measurements and laboratory analyses. Temperature (T), pH, redox potential (ORP), dissolved oxygen (DO), and electrical conductivity (EC) were measured on-site using a portable water quality analyzer (CLEAN, Shanghai ZhenMai Instruments, Shanghai, China). The measurement accuracies of the portable devices were as follows: ±0.1 °C for T, ±0.01 for pH, ±1 mV for ORP, ±0.1 mg/L for DO, and ±0.1 μS/cm for EC [12,14]. Due to the potential variability of NO_3_^−^ transformation, its concentration was measured in the field using a portable spectrophotometer (DR1900, HACH, Loveland, CO, USA) following the manufacturer’s instructions, with a detection limit of 0.08 mg/L [14]. Major cations (Na^+^, K^+^, Ca^2+^, and Mg^2+^) were quantified by Inductively Coupled Plasma Optical Emission Spectrometry (ICP-OES, SPECTRO Analytical Instruments, Kleve, Germany); Cl^−^ and SO_4_^2−^ were measured using Ion Chromatography (ICS-90, Dionex Thermo Scientific, Sunnyvale, CA, USA); HCO_3_^−^ was determined by acid-base titration using an auto-analyzer (905 Titrando, Metrohm AG, Herisau, Switzerland) [14]. The measurement precision for HCO_3_^−^ was 0.6 mg/L, and the analytical precision for other hydrochemical variables were 0.01 mg/L [14]. Total dissolved solids (TDS) were calculated as the sum of major ions minus half of the HCO_3_^−^ concentration.

### 2.3. DNA Extraction, PCR Amplification, and Bioinformatics Treatment

Genomic DNA retained in the filter membrane was extracted using FastDNA™ SPIN Kit for Soil (MP Biomedicals, Santa Ana, CA, USA) according to the manufacturer’s instructions. The purity and concentration of the DNA were assessed using Nanodrop 2000 (Thermo Fisher Scientific, Waltham, MA, USA) [9]. Polymerase chain reaction (PCR) was performed to amplify V4 hypervariable region of 16S rRNA genes using general primer set of 515F (5′-GTGCCAGCMGCCGCGG-3′) and 806R (5′-GGACTACHVGGGTWTCTAAT-3′) [17]. PCR program consisted of initial denaturation at 95 °C for 5 min, 29 cycles of 95 °C (30 s), 52 °C (30 s), and 72 °C (45 s) and a final extension at 72 °C for 10 min. PCR amplification was performed using a BioRad S1000 Thermal Cycler (Bio-Rad Laboratory, Hercules, CA, USA) [18]. The PCR amplicons were verified by electrophoresis on a 1.5% (wt/vol) agarose gel. After confirming the main bands fell within the normal range, PCR products from different samples were mixed in equal mass proportions. Additionally, it should be noted that the primers used for PCR amplification contain sample-specific barcodes, which are crucial for distinguishing different samples during subsequent sequencing and data analysis [12]. The mixed products were recovered using the E.Z.N.A.^®^ Gel Extraction Kit (Omega, Norcross, GA, USA) for gel recovery, with target DNA fragments eluted in TE buffer [19]. Library construction is carried out following the standard protocol of the ALFA-SEQ DNA Library Prep Kit, and the size of the library fragments is evaluated on the Qsep 400 High-Throughput Nucleic Acid and Protein Analysis System (Hangzhou Houze Biotechnology Co., Ltd., Hangzhou, China). The concentration of the library is measured using Qubit 4.0 (Thermo Fisher Scientific, Waltham, MA, USA). The constructed amplicon libraries are subjected to PE250 sequencing on the Illumina platform (Guangdong Magigene Biotechnology Co., Ltd. Guangzhou, China).

The paired-end raw reads underwent initial processing using fastp (v0.14.1) with sliding-window quality trimming parameters (-W 4 -M 20), followed by adapter and primer removal via Cutadapt to yield Paired-end Clean Reads. These clean reads were then merged into Raw Tags based on sequence overlaps using the USEARCH fastq_mergepairs module. Subsequent re-application of identical fastp (-W 4 -M 20) trimming parameters generated the final Clean Tags. Operational Taxonomic Units (OTUs) were clustered at 97% similarity from the Clean Tags using the UPARSE algorithm. Representative sequences from each OTU were taxonomically annotated through alignment against the SILVA SSU115 reference database for preliminary classification, with further refinement and enhanced reliability achieved by querying the NCBI taxonomy database [20,21].

### 2.4. Statistical Analysis and Modeling

Due to limitations in the use of field-testing equipment, all high-salinity groundwater samples are missing T, EC, DO, and ORP parameters. Some artesian groundwater samples lack DO, ORP, and NO_3_^−^ data, and pH is missing for sample HSw3-15. Therefore, the “mice” package in R was used to construct a multiple imputation model based on other hydrochemical parameters of the samples to fill in the missing data for subsequent multivariate statistical analysis [22]. Correlations between hydrochemical variables were assessed using Spearman’s correlation coefficient [23]. Principal Component Analysis (PCA) was employed to reduce dimensionality by projecting multivariate data into a lower-dimensional space, while maximizing the preservation of variance from the original dataset. This approach allows for an effective visualization of similarities and differences among sampling sites in terms of hydrochemical properties and microbial community composition [24]. Distance matrices were generated using the Euclidean Distance and Bray–Curtis Distance algorithms, respectively, to quantify the similarity and dissimilarity in hydrochemical properties and microbial community composition between samples [25,26]. The significance of differences in hydrochemical characteristics and microbial communities under varying conditions was assessed using the Kruskal–Wallis test (SPSS 26, IBM Company, Armonk, NY, USA, 2010) [27].

Redundancy Analysis (RDA) was employed to quantify the influence of environmental factors on microbial community structure and to identify key environmental variables and their respective contributions [28]. To address statistical bias induced by multicollinearity among conventional variables, a hierarchical partitioning algorithm was applied to optimize the assessment of the contributions of multi-dimensional environmental variables to microbial community variation [29]. Partial Least Squares Structural Equation Modeling (PLS-SEM) was employed to unravel the complex causal networks between multi-dimensional environmental factors and underlying ecological processes. PLS-SEM integrates a dual analytical framework, including measurement and structural models. The measurement model validates the loadings between observed variables and latent variables, while the structural model quantifies both the direct path effects and indirect cascading effects among latent variables [30]. The algorithm is based on an iterative least square fitting approach, which overcomes the limitations of traditional covariance-based structural equation modeling regarding data normality and large sample requirements.

Microbial co-occurrence networks were constructed using the iNAP platform based on Hellinger-transformed OTU abundance tables to elucidate interspecies relations [31]. OTUs within the top 50%, 40%, and 50% abundance were selected for phreatic, artesian, and high-salinity samples, respectively. Species correlations were computed using SparCC, and topological parameters were computed via the iNAP platform. Keystone taxa were identified based on hub score (Zi > 2.5) and connector score (Pi > 0.62). Network visualization was performed in Gephi (v0.10.1) using the Fruchterman–Reingold layout algorithm [31].

The Beta Nearest Taxon Index (βNTI) is a phylogenetics-based metric designed to quantify the relative contributions of deterministic processes (e.g., environmental selection) and stochastic processes (e.g., dispersal limitation, random birth–death event) in microbial community assembly. It works by comparing the observed phylogenetic turnover between communities with that expected under a null model, thereby revealing the underlying mechanisms driving community succession or spatial distribution [7]. Microbial community assembly processes were inferred based on βNTI values. A βNTI < −2 indicates homogeneous selection driven by environmental filtering, leading to community convergence in similar environments. A βNTI > +2 suggests heterogeneous selection caused by environmental heterogeneity, resulting in community divergence. For |βNTI| ≤ 2, stochastic processes are considered dominant and require additional calculation of the Raup–Crick index (RC_bray_) from Bray–Curtis dissimilarity matrices using a null model. This model partitions stochasticity into dispersal limitation, homogenizing dispersal, and ecological drift. RC_bray_ > +0.95 indicates dispersal limitation, RC_bray_ < –0.95 indicates homogenizing dispersal, and |RC_bray_| ≤ 0.95 suggests undominated processes, likely driven by ecological drift or mixed stochastic factors [6,7]. To further quantify the relative contributions of deterministic and stochastic processes, the Normalized Stochasticity Ratio (NST) was computed using the “NST package” in R. This metric estimates the proportion of stochasticity by comparing observed community β-diversity (Bray–Curtis dissimilarity) with values predicted by deterministic models and expected under null models [32]. NST values ≤ 50% indicate deterministic processes dominate, values between 50% and 80% suggest a combination of deterministic and stochastic influences, and values > 80% reflect a predominance of stochastic processes.

## 3. Results and Discussion

### 3.1. Regional Hydrochemical Characteristics

Groundwater hydrochemical parameters exhibited pronounced spatial patterns regardless of sampling time. Along the groundwater flow path, remarkable differences in hydrochemistry were observed among phreatic water, artesian water, and high-salinity groundwater (Table 1). DO declined from 4.61 ± 0.54 mg/L in phreatic groundwater to 3.29 ± 0.32 mg/L in artesian groundwater, representing a 28.57% decrease. Similarly, ORP decreased from 176.42 ± 24.61 mV to 60.95 ± 18.11 mV, corresponding to a 65.45% reduction. These trends indicate a gradual transition from oxidizing to reducing conditions along the groundwater flow path, reflecting the progressive depletion of dissolved oxygen through a series of complex physical, chemical, and biological processes within the aquifer systems since groundwater recharge, such as microbially mediated redox reactions, mineral dissolution and precipitation, and ion exchange [33,34]. Groundwater pH gradually evolved from a slightly alkaline environment in phreatic groundwater (8.00 ± 0.04) and artesian groundwater (8.04 ± 0.02) towards a neutral to acidic environment in high-salinity groundwater (6.63 ± 0.03), potentially influenced by carbonate equilibrium and microbially mediated oxidative processes [35].

**Figure 2:**
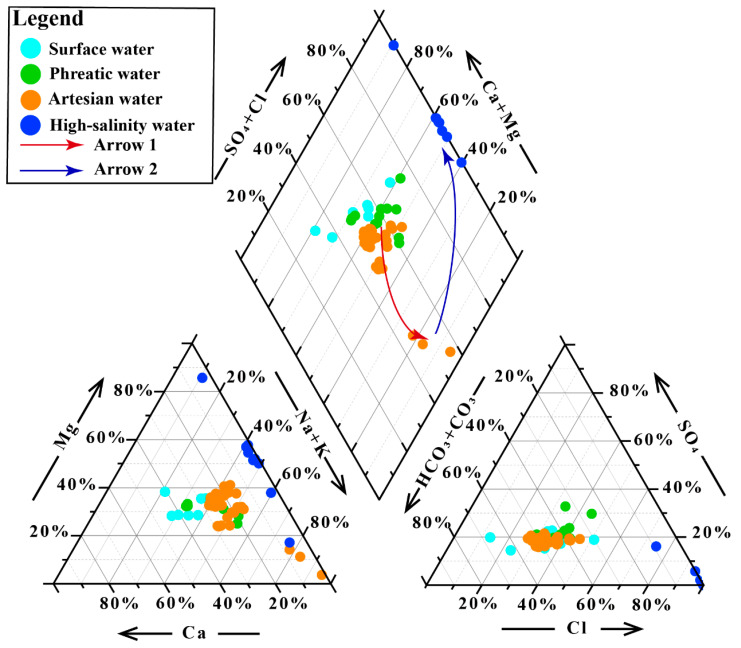
Piper diagram of hydrochemical samples.

Major ionic constituents in groundwater exhibited a two-stage evolutionary trend (Figure 2). TDS displayed minimal variations during the groundwater flow from the phreatic to the confined aquifer, characterized mainly by the relative enrichment of Na^+^ and K^+^ (Arrow 1, Figure 2). In contrast, the transition from artesian groundwater to high-salinity groundwater was marked by a sharp increase in ion concentrations (TDS of artesian water: 486.01 ± 16.83 mg/L; high-salinity groundwater: 316,112.74 ± 12,452.19 mg/L), with the hydrochemical composition dominated by the enrichment of Cl^−^ and Mg^2+^ (Arrow 2, Figure 2). The increase in major ions is primarily controlled by water–rock interactions and evaporative concentration processes, with the occurrence of high-salinity groundwater closely linked to evaporative concentration under extreme arid conditions [14,36]. Na^+^, K^+^, Mg^2+^, and Ca^2+^ all showed an initial slow increase followed by rapid accumulation along the groundwater flow path. Among them, Ca^2+^ accumulated at a much slower rate compared to the other cations, likely due to carbonate solubility controls, which is consistent with the observed variations in HCO_3_^−^. Cl^−^ showed a similar variation pattern to the cations, whereas SO_4_^2−^ displayed an initial increase followed by a decrease, largely related to sulfate reduction under reducing conditions in confined aquifers [37]. Additionally, the river water exhibited a similar hydrochemical evolutionary path consistent with that of groundwater (Figure 2).

**Figure 3:**
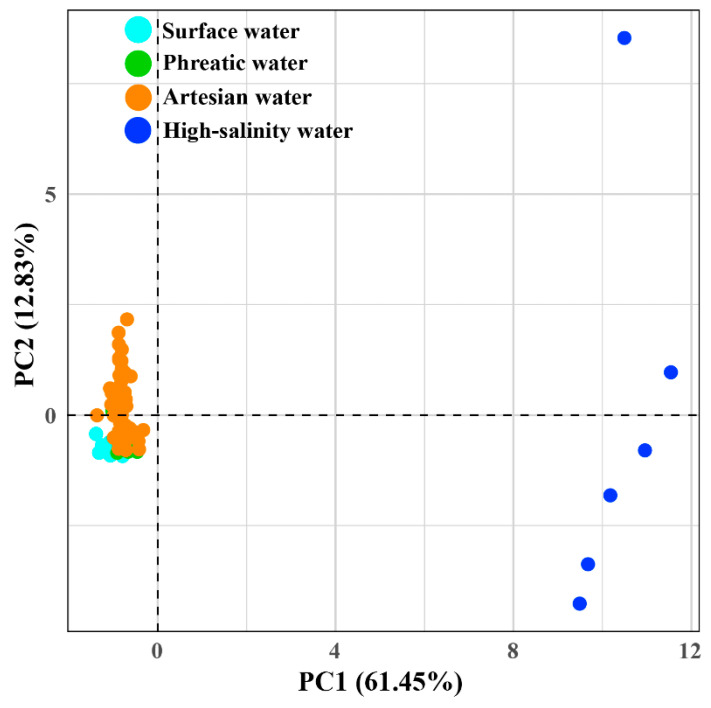
PCA biplot of hydrochemical data to identify hydrochemical characteristics.

PCA extracted four components with eigenvalues greater than 1 (PC1 to PC4) based on 14 hydrochemical variables, which collectively explained 90.43% of the total variance (Figure 3, Table S2). PC1 was composed of major ions (Na^+^, K^+^, Ca^2+^, Mg^2+^, Cl^−^, SO_4_^2−^, HCO_3_^−^, and NO_3_^−^) and accounted for 61.45% of the total variance. The two-dimensional plot indicates that PC1 mainly drives the separation between low-TDS groundwater and high-salinity groundwater (*p* < 0.05, Figure S1), highlighting substantial differences resulting from ion enrichment driven by evaporative concentration [14]. PC2 and PC3 explained 12.83% and 8.97% of the total variance, respectively. PC2 was primarily influenced by ORP, whereas PC3 was mainly associated with DO (Table 2). Together, they characterize the redox variance along the groundwater flow path, illustrating redox conditions as the second most important factor in the process of hydrochemical evolution. PC4 accounted for 7.19% of the total variance, with T (loading 0.85) as the primary associated factor, representing the influence of water temperature.

Although the extreme hydrochemical constitution of high-salinity groundwater causes overlaps between low-TDS groundwater and river samples in Figure 3, the PCA results after excluding high-salinity groundwater still reveal remarkable hydrochemical differences between river water, phreatic groundwater, and artesian groundwater (Figure S2).

### 3.2. Microbial Alpha-Diversity and Composition Along Groundwater Flow Path

A total of 5,561,433 valid sequences were obtained from the high-throughput sequencing of microbial samples and clustered into 20,384 OTUs after sequence alignment. The rank–abundance curve indicated that the sequencing depth was sufficient to capture nearly the full extent of the groundwater microbial community (Figure S3). In this study, α-diversity indices were used to assess microbial community diversity (Table 3, Figure S4). The results revealed a decreasing trend in microbial richness along the groundwater flow path. In phreatic groundwater, the Sobs index was 974.83 ± 208.93, which declined to 756.84 ± 118.67 in artesian groundwater and 811.75 ± 111.73 in high-salinity groundwater. A similar pattern was observed for the ace index, which declined from 1197 ± 249.97 in phreatic groundwater to 958.57 ± 144.91 in artesian groundwater and 1028.5 ± 188.52 in high-salinity groundwater. The Shannon index of microbial communities in phreatic groundwater was 3.88 ± 0.26, which decreased by 6.18% and 9.34% in artesian groundwater (3.64 ± 0.15) and high-salinity groundwater (3.52 ± 0.34), respectively. In contrast, the Simpson index increased from 0.081 ± 0.01 in phreatic groundwater to 0.096 ± 0.01 in artesian groundwater and 0.108 ± 0.03 in high-salinity groundwater, collectively indicating a gradual decline in microbial diversity. The Pielou_e index reflects the evenness of species distribution within the inner community. Similar Pielou_e values were observed in phreatic and artesian groundwater (0.593 ± 0.03 and 0.596 ± 0.02, respectively). However, in high-salinity groundwater, the evenness declined (0.536 ± 0.05), likely due to environmental stress eliminating saline-sensitive species and increasing the relative abundance of halotolerant ones. Overall, the α-diversity indices reveal significant shifts in microbial communities across different groundwater environments. Along the groundwater flow path, the number and abundance of species in microbial communities gradually decrease, while the relative proportion of dominant species increases, suggesting that changes in hydrochemical conditions may be the primary driver of microbial communities. Notably, river water samples exhibited higher richness (3757.60 ± 719.98 in Sobs, 4666 ± 735.71 in ace), diversity (5.20 ± 0.65 in Shannon), and evenness (0.693 ± 0.06 in Pielou_e) than groundwater. This is closely associated with the dynamic flow of the river, the diverse aquatic habitats, and the continuous input of terrestrial microorganisms [38,39].

**Figure 4:**
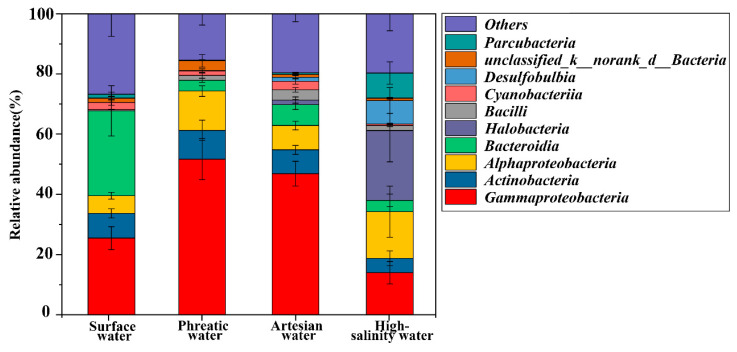
Microbial community composition at the class level.

Microbial community compositions varied across different groundwater environments. *Gammaproteobacteria*, *Alphaproteobacteria*, *Actinobacteria*, and *Halobacteria* were dominant taxa in groundwater, totally accounting for more than 65% of the microbial community (Figure 4, Table S3). *Gammaproteobacteria* exhibited the highest abundance in subsurface aquatic environments, decreasing from 51.69 ± 6.83% in phreatic groundwater to 46.84 ± 4.11% in artesian groundwater, and further down to 13.97 ± 3.70% in high-salinity groundwater. This variation may be attributed to the ecological preference of *Gammaproteobacteria* for freshwater environments. In contrast, hypersaline conditions disrupt the osmotic balance of the cells of these microbial taxa, leading to cellular dehydration and, consequently, a decrease in their relative abundance within the microbial community [40]. *Alphaproteobacteria* showed a similar declining trend in phreatic and artesian groundwater, with relative abundances of 13.06 ± 1.79% and 8.08 ± 1.45%, respectively. However, the relative abundance of *Alphaproteobacteria* increased to 15.48 ± 8.47% in high-salinity groundwater, largely due to an increase in *Rhodobacteraceae*, a family within *Alphaproteobacteria* known for its salinity tolerance, which was capable of thriving under hypersaline conditions (Table S4) [41]. *Actinobacteria* accounted for 9.54 ± 3.40% and 7.93 ± 1.48% in phreatic and artesian groundwater but lowered to 4.77 ± 2.46% in high-salinity groundwater. Notably, the relative abundance of halophilic microbes such as *Halobacteria* and *Parcubacteria* increased significantly in high-salinity groundwater. In low-TDS groundwater, *Halobacteria* and *Parcubacteria* were seldom detected with very low abundance (0.01 ± 0.004% and 0.28 ± 0.09% in phreatic groundwater, 1.50 ± 1.03% and 0.50 ± 0.15% in artesian groundwater), but their abundances rose sharply to 23.22 ± 10.42% and 8.34 ± 3.71%, respectively, in high-salinity groundwater. In such environments, halophiles like *Halobacteria* depend on Na^+^/H^+^ antiporters and ATPase-powered ion pumps to actively accumulate high internal levels of K^+^ and Cl^−^, enabling osmotic balance with the hypersaline surroundings and supporting normal cellular functions [42,43]. In addition, *Bacteroidia* showed a significantly higher abundance in river water at 28.15 ± 8.38%, compared to 3.56 ± 0.71% in phreatic groundwater, 6.95 ± 1.63% in artesian groundwater, and 3.73 ± 1.97% in high-salinity groundwater (*p* < 0.05). These findings further indicate that shifts in species composition are closely linked to hydrochemical conditions, reflecting the impacts of environmental filtering.

**Figure 5:**
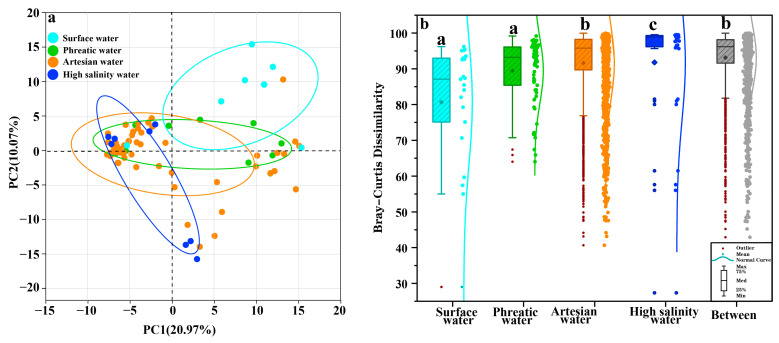
PCA biplot of hydrochemical data to identify hydrochemical characteristics (**a**); box plots show differences in microbial community structure across sample types (**b**).

Similarly to hydrochemical analysis, PCA was used to extract overall structural features of microbial communities, revealing distinct differences among microbial communities across hydrochemical environments along the groundwater flow path (Figure 5a). Microbial communities from phreatic and artesian groundwater were scattered along the horizontal axis, while those from high-salinity environments formed a larger angle with both horizontal (20.97%) and vertical axes (10.07%), supporting previous findings regarding distinct community structures. The results from the Kruskal–Wallis test confirmed significant distinctions in microbial community structures among phreatic groundwater, artesian groundwater, and high-salinity groundwater environments (*p* < 0.05, Figure 5b), suggesting that hydrochemical conditions may serve as a key selective factor driving microbial community differentiation. The distinct evolution mechanisms and dominant factors across groundwater hydrochemical environments are likely the main driving forces for the differentiated microbial community structures. Due to the relatively similar hydrochemical characteristics of river water and phreatic groundwater, there was no significant difference in their microbial communities (*p* > 0.05, Figure 5b).

### 3.3. Driven Forces of Microbial Assemblages

**Figure 6:**
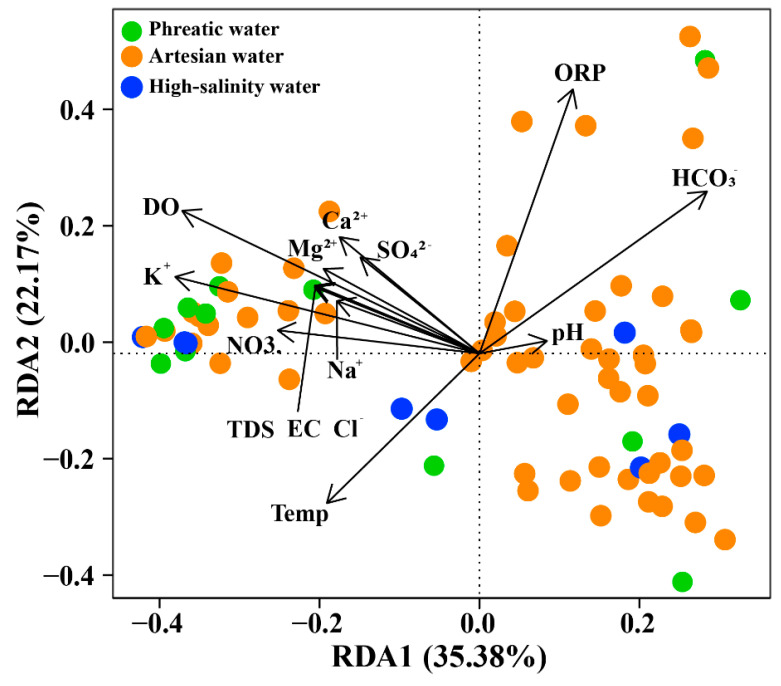
RDA ordination of microbial communities at the Class level.

In this study, RDA combined with hierarchical partitioning was used to quantify the influence and relative contributions of environmental factors on microbial community structure, while effectively minimizing the interference of multicollinearity among variables [44,45]. This approach performs a global decomposition of all possible subsets of variables and quantifies the importance of each variable by calculating its independent and shared contributions across different combinations (Figure 6, Table S5). The results showed that hydrochemical environments had a significant impact on microbial communities. RDA1 and RDA2 explained over 35.38% and 22.17% of the total variance in community composition, respectively. Hydrochemical variables, including K^+^, Na^+^, DO, pH, and EC, contributed substantially to the total microbial variance (individual percentage > 10%; Table S5). K^+^, Na^+^, and EC are representative variables of the salinity accumulation process in groundwater, as shown in the PCA results, reflecting the hydrochemical evolution from phreatic to artesian and ultimately to high-salinity groundwater (Figure 3, Table 2). Hierarchical partitioning results showed that these representative variables contributed most to the variations in microbial communities, highlighting the controlling effect of salinity accumulation in shaping microbial community structure. Salinity accumulation leads to cellular dehydration, forcing microorganisms to maintain intracellular homeostasis by accumulating compatible solutes or actively pumping out ions. Such hydrochemical conditions align with the ecological preference of halophiles, which is consistent with the high abundance of halophilic microorganisms observed in high-salinity groundwater [46]. DO is a key factor influencing the redox condition of groundwater, particularly under oxic conditions. As the most energy-efficient electron acceptor, the availability of DO determines microbial respiratory pathways, with microorganisms preferentially carrying out aerobic respiration and switching to alternative electron acceptors only when DO becomes limited [47]. In the study area, DO decreases by 28.57% from phreatic to artesian groundwater, while ORP drops by 65.45%, indicating a shift from oxidizing to reducing conditions. Such a decline in DO concentration indicates increasing difficulty in the acquisition of electron acceptors, triggering shifts in microbial respiratory strategies, as evidenced by the decreased abundance of aerobic or facultative aerobic taxa like *Alphaproteobacteria* and *Actinobacteria*, alongside a corresponding increase in anaerobic groups such as *Desulfobulbia* [48]. Notably, ORP exerts a similar influence on microbial community composition. This collectively highlights how DO and ORP variations drive microbial metabolic adaptation.

To further identify the primary factors controlling microbial communities in phreatic, artesian, and high-salinity groundwater, three PLS-SEM models were constructed. These models integrated both hydrochemical characteristics and geographic distribution to assess their combined effects on microbial community structure. In considering hydrochemical characteristics and their evolutionary processes, the latent variables Major Hydrochemical Ions (MHIs) and Redox-Physical Parameters (RPPs) were constructed to represent the hydrochemical effects in the model. The latent variable MHIs was represented by four observed hydrochemical variables: Na^+^, Ca^2+^, Mg^2+^, and SO_4_^2−^, while the other latent variable, RPPs, was defined by DO, ORP, and T. Given the pronounced changes in hydrochemical composition along the groundwater flow path, longitude and latitude were also introduced as observed variables to represent the latent variable Space, enabling the model to assess spatial influences on hydrochemical parameters and microbial communities. On the other hand, microbial communities were represented by a set of α- and β-diversity indices, which were grouped under the latent variable Community.

**Figure 7:**
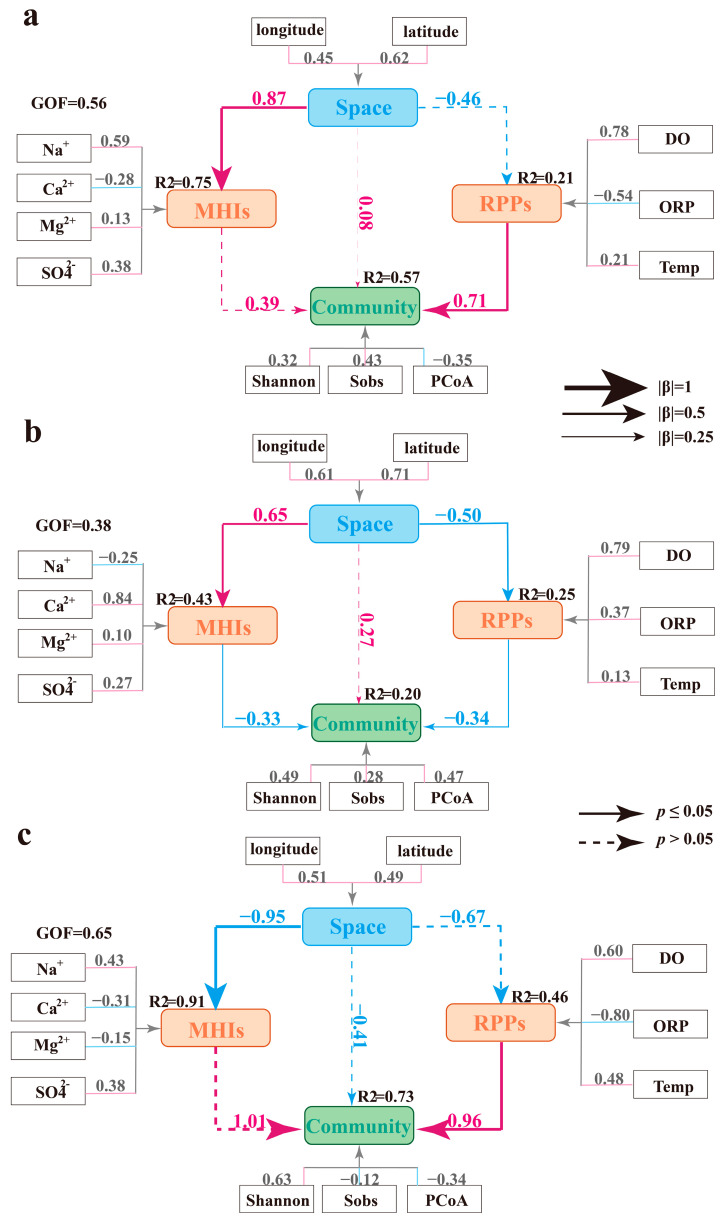
PLS-SEM models illustrating the environmental drivers of microbial community structure across phreatic groundwater (**a**), artesian groundwater (**b**), and high-salinity groundwater environments (**c**).

All three models showed satisfactory fitness, with GOF values of 0.56, 0.38, and 0.65, respectively, meeting the PLS-SEM requirements (Figure 7). The modeling results indicated that groundwater microbial communities were primarily driven by hydrochemical conditions, with the dominant influencing factors varying across different groundwater environments. In contrast, the spatial distribution of microbial communities within the groundwater flow system showed no significant effect on microbial community structure (Figure 7). In the PLS-SEM of phreatic groundwater, the microbial community was primarily driven by the latent variable RPPs (total effect (β) = 0.71, *p* < 0.05), with DO showing the highest loading (0.78), indicating its crucial regulatory effect in shaping microbial communities. This is consistent with the observed dominance of aerobic or facultative aerobic taxa, such as *Alphaproteobacteria* and *Actinobacteria*, in the phreatic microbial communities. In contrast, MHIs showed close relations with sampling location, and due to the relatively small variation in major ions in phreatic groundwater latent variables, MHIs, and Space had limited effects on the microbial community (*p* > 0.05, Figure 7a). In artesian groundwater, both MHIs and RPPs exhibited clear trends of variation: major ions began to gradually accumulate along the groundwater flow path, while DO and ORP showed significant decreases (*p* < 0.05), consistent with the previous hydrochemical analysis. PLS-SEM results indicated that both latent variables, MHIs and RPPs, had significant effects on the structure of microbial communities in artesian groundwater (Figure 7b). The emerging salinity–oxygen stress in the hydrochemical environment was identified as the main driving force behind microbial community shifts (β = −0.34, *p* < 0.05). In high-salinity environments, MHIs were elevated to extremely high levels, and the groundwater’s ionic composition exhibited clear spatial distributions. Salinity stress in these environments significantly altered the microbial community structure, with halophilic taxa such as *Halobacteria* and *Parcubacteria* becoming dominant, highlighting the strong link between microbial communities and hydrochemical characteristics (β = 1.01, *p* > 0.05, Figure 7c). In addition, RPPs showed a strong association with the microbial community structure, even though direct measurements of DO, ORP, and T were unavailable in high-salinity samples. Calculated data inferred from hydrochemical trends still demonstrated the strong influence of RPPs on microbial communities (β = 0.96, *p* < 0.05). We speculate that high-salinity groundwater is likely characterized by low-oxygen and reducing conditions, and the PLS-SEM results further indicate that microbial communities are sensitive to fluctuations in DO, ORP, and T [49].

### 3.4. Microbial Co-Occurrence Network

**Figure 8:**
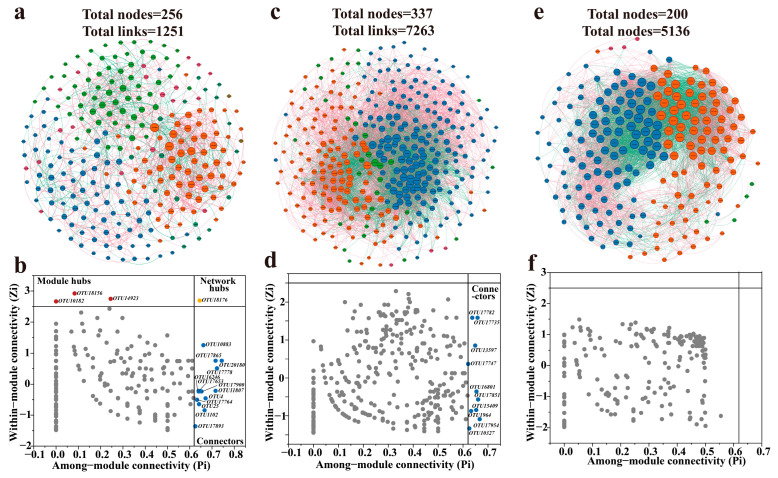
Microbial co-occurrence networks and the corresponding results about the roles of node-level in networks across phreatic (**a**,**b**), artesian (**c**,**d**), and high-salinity (**e**,**f**) groundwater environments. The roles of nodes in co-occurrence networks are indicated by their values of within-module connectivity (Zi) and among-module connectivity (Pi).

The above analysis investigated the hydrochemical characteristics and microbial community structures of groundwater and identified the driven forces of microbial communities integrating RDA, hierarchical partitioning, and PLS-SEM approaches. However, the interspecies relationships within different groundwater environments remain unclear. To address this, microbial co-occurrence networks were employed to further explore the connections and distinctions in community composition among phreatic, artesian, and high-salinity groundwater environments. The microbial co-occurrence networks revealed that the microbial community in phreatic groundwater was composed of six modules (Q = 0.507, 256 nodes, 1251 edges), while the artesian groundwater community consisted of four major modules (Q = 0.255, 337 nodes, 7265 edges), and the high-salinity groundwater community included four modules as well (Q = 0.244, 200 nodes, 5136 edges) (Table S6). The number of modules reflects relatively cohesive community compositions. Phreatic groundwater exhibited a greater number of modules compared to artesian and high-salinity groundwater environments, suggesting a trend toward more centralized microbial community structures as groundwater environments evolve (Figure 8).

The microbial network in phreatic groundwater had the lowest density (0.04) and average degree (avgK, 9.77) among the three environments, while exhibiting the highest average path distance (GD, 3.33), suggesting a more loosely connected network structure with sparse links and longer average distances between species (Figure 8a). This network topology suggests that high modularity is often associated with low connectivity density and low average degree. Previous studies have also reported that higher GD values indicate longer information transmission pathways, slower response rates, and reduced community stability; the phreatic network’s lowest geodesic efficiency (0.34) further supports this interpretation [50,51]. In addition, the phreatic microbial community exhibited the highest proportion of positive correlations among species (65.07%), which is closely related to its occurrence in a relatively resource-rich recharge environment. The high oxygen content and low competitive pressure in phreatic groundwater favor cooperative and coexistent interactions among microbial taxa [52]. According to the Zi-Pi plot, the phreatic microbial network contained three module hubs, one network hub, and thirteen connectors (Figure 8b). *Polaromonas* (OTU10182), *Methylobacterium-Methylorubrum* (OTU18156), and *Woesearchaeales* (OTU14923) were identified as module hubs, all known for their high functional metabolic flexibility and critical ecological roles [53,54,55]. A total of 13 OTUs served as connectors within the community, belonging to *Actinobacteria*, *Bacilli*, *Alphaproteobacteria*, *Vicinamibacteria*, *Gammaproteobacteria*. These lineages often act in coordination within microbial ecosystems to degrade organic matter and participate in biogeochemical cycling, collectively maintaining microbial network balance and function [56,57].

In the artesian groundwater, the microbial co-occurrence network exhibited the largest scale among all groundwater types, comprising 337 nodes and 7265 edges (Figure 8c). The network density (0.13) and average degree (avgK, 43.10) were significantly higher than those of the phreatic groundwater (density: 0.038, avgK: 9.77). Moreover, the reduced average path distance (GD, 2.22) and elevated geodesic efficiency (0.51) together indicate that the microbial community in artesian groundwater has evolved into a highly interconnected and efficient network, characterized by strengthened interspecies interactions. As groundwater flows into the confined aquifer, hydrochemical variations, including sustained depletion of DO, decreased ORP, and enrichment of Na^+^ and K^+^, intensify microbial competition, leading to an increase in the proportion of negative correlations to 39.93% and ecological niche differentiation. Nevertheless, positive correlations (60.07%) remained dominant. Microbial species with relatively high abundances, such as *Gammaproteobacteria* (OTU17851), *Bacteroidia* (OTU17954), and *Desulfovibrionia* (OTU17735), were also observed as network connectors. Compared to phreatic groundwater, the microbial community in the artesian environment exhibited a tighter network of associations, reflecting that microbes in closed groundwater systems rely on key connectors to establish close symbiotic relationships in response to environmental changes (Figure 8d) [58].

The microbial co-occurrence network in high-salinity groundwater consisted of 4 modules, 200 nodes, and 5136 edges (Figure 8e). Although the number of nodes and edges was lower than that in the artesian groundwater environment, the network exhibited the highest density (0.26) and average degree (avgK, 51.63) among all groundwater types, indicating a highly interconnected community. This tightly linked co-occurrence network likely serves as an adaptive strategy to cope with high salinity stress. Negative correlations accounted for 43.33% of the network edges, the highest proportion observed across groundwater environments, indicating intensified microbial competition under saline stress. This is further supported by the shortest average path distance (GD, 2.07) and the highest geodesic efficiency (0.58), which together reflect enhanced information transfer and tighter microbial competitive interactions in high-salinity conditions. These features imply that microbial communities in high-salinity groundwater are shaped by intensified competition, resulting in a closely interconnected network structure. However, the combined effects of high salinity and strong competitive pressure suppressed the emergence of potential connectors, with all species identified as peripheral species (Figure 8f), indicating intensified interactions within modules and reduced demand for cross-module connectivity [59].

### 3.5. Microbial Community Assembly in Different Hydrochemical Conditions

**Figure 9:**
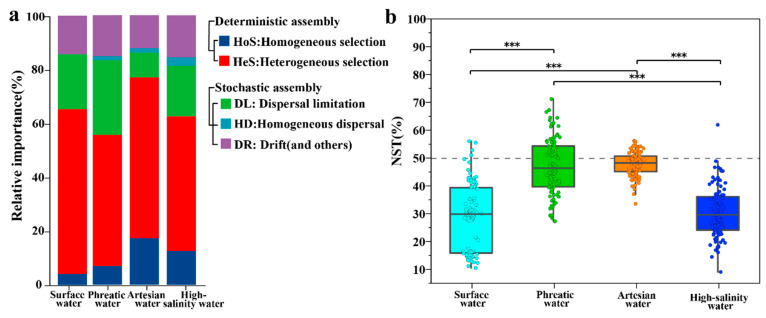
βNTI analysis of microbial community assembly processes (**a**), and box plot comparison of NST (**b**) across distinct water types (*** represents *p* < 0.001).

Microbial communities in groundwater are mainly shaped by hydrochemical features and their evolutionary processes, while the impact of geographic spatial distribution on community patterns seems to be minimal. We employed a null model analysis based on the β-nearest taxon index (βNTI) to quantify the relative contributions of deterministic and stochastic processes in microbial community assembly. The results revealed that deterministic processes (|βNTI| > 2) play a dominant role in shaping microbial communities in groundwater environments (Figure 9a, Table S7), consistent with the previous findings.

Among deterministic processes, heterogeneous selection (βNTI > +2) exerted the most significant influence, accounting for 48.61%, 59.63%, and 50.00% of community assembly in phreatic groundwater, artesian groundwater, and high-salinity groundwater, respectively. This highlights the central role of environmental heterogeneity in driving microbial community assemblies. On the other hand, homogeneous selection (βNTI < −2) contributed less overall, with proportions of 6.94%, 17.28%, and 12.50% in the three groundwater environments, respectively. Notably, the relatively higher proportions of homogeneous selection observed in artesian groundwater (17.28%) and high-salinity groundwater (12.50%) suggest that environmental homogenization in closed subsurface systems promotes community convergence. Similarly to groundwater, microbial communities in rivers were also dominated by deterministic processes, with heterogeneous and homogeneous selection accounting for 61.22% and 4.08%, respectively. Overall, deterministic selection governs the formation of groundwater microbial community structures, forming the ecological assembly framework under salinity and redox stress.

Despite the dominance of deterministic processes, stochastic processes (|βNTI| < 2) still accounted for a substantial proportion of community assembly (ranging from 23.09% to 44.44%) across the groundwater environment. These processes are more closely related to microbial life-history traits rather than environmental filtering. Dispersal limitation was the second most important process (RC_bray_ > +0.95), with varying contributions across groundwater types: phreatic groundwater showed the highest proportion (27.78%), followed by high-salinity groundwater (18.75%), and artesian groundwater (9.18%). The higher contribution in phreatic groundwater is likely due to its more active exchange with surface water, which introduces randomness as recharge occurs through heterogeneous vadose zones [14]. Drift and other ecological processes (12.31–15.63%) were the second most important components among different stochastic processes, suggesting that random factors such as gene transfer and species turnover also play a role in groundwater microbial community assembly [7].

Further validation through the normalized stochasticity ratio (NST) supported the results of the βNTI findings. NST values were all below 50% across hydrochemical environments, specifically 47.19 ± 2.87% for phreatic groundwater, 48.20 ± 0.58% for artesian groundwater, and 30.75 ± 3.12% for high-salinity groundwater, as well as 29.36 ± 4.44% for river water (Figure 9b), indicating deterministic processes predominantly govern microbial community assembly. The significantly lower NST value in high-salinity groundwater compared to phreatic and artesian groundwater (*p* < 0.05) underscores the decisive role of salinity–redox stress in shaping assembly mechanisms. Together with the high contribution of heterogeneous selection, these results collectively indicate that environmental conditions serve as universal drivers of microbial community assembly across groundwater environments [7]. Although dispersal limitation, homogeneous dispersal, and homogeneous selection may have some effects in specific groundwater environments, environmental filtering remains the primary driver across all aquatic environments. The absolute dominance of heterogeneous selection emphasizes the fundamental role of environmental heterogeneity in structuring groundwater microbial communities.

## 4. Conclusions

This study systematically investigated the microbial community assembly processes and the driving forces along the groundwater flow path in the Golmud River watershed. The findings provide deeper insight into how hydrochemical processes shape groundwater microbial assemblages, particularly under salinity–oxygen stress. The main conclusions are as follows:

1. Hydrochemical conditions shifted from oxidizing to reducing environments along the groundwater flow path, while major ions exhibit minimal variations from the phreatic groundwater to the artesian groundwater, followed by pronounced enrichment during the evolution from artesian groundwater to high-salinity groundwater.

2. Decreased microbial alpha diversities were observed from phreatic groundwater to high-salinity groundwater. The relative abundance of predominant microorganisms such as *Gammaproteobacteria* and *Actinobacteria* declined across phreatic, artesian, and high-salinity groundwater, while halophiles such as *Halobacteria* and *Parcubacteria* emerged as the most abundant lineages in high-salinity environments.

3. Deterministic processes controlled microbial community assembly across different hydrochemical conditions. Microbial communities were all governed by RPPs, while MHIs had a close correlation with microbial communities in artesian and high-salinity groundwater. Microbial co-occurrence networks revealed increasingly tight interactions and intensified competition among communities, driven by accumulated salinity–oxygen stress along the groundwater flow path.

## Figures and Tables

**Table 1:** The descriptive statistics of hydrochemical data for different sample types.

	Statistic	pH	T	EC	DO	ORP	TDS	Na^+^	K^+^	Ca^2+^	Mg^2+^	Cl^−^	SO_4_^2−^	HCO_3_^−^	NO_3_^−^
			°C	μs/cm	mg/L	mv	mg/L	mg/L	mg/L	mg/L	mg/L	mg/L	mg/L	mg/L	mg/L
Phreaticz water	*n* ^a^	12	12	12	12	12	12	12	12	12	12	12	12	12	12
	Max	8.23	22.40	1300.00	9.54	280.00	755.03	122.00	5.38	68.30	54.70	209.00	188.00	241.00	4.87
	Min	7.70	9.30	775.00	2.01	9.00	413.07	55.20	2.33	38.70	28.00	74.95	66.66	196.47	2.54
	Mean	8.00	13.21	938.08	4.61	176.42	519.72	89.11	4.04	50.40	37.34	120.38	102.28	224.33	3.79
	SE ^b^	0.04	1.00	43.12	0.54	24.61	27.71	6.62	0.29	2.45	1.93	10.21	10.99	4.12	0.19
Artesian water	*n*	56	56	56	56	56	56	56	56	56	56	56	56	56	48
	Max	8.65	23.40	1485.00	8.29	272.00	1048.00	374.08	7.00	75.80	44.12	200.56	139.96	404.77	6.16
	Min	7.75	6.80	659.00	0.39	−234	383.14	64.80	2.68	4.93	7.77	79.70	54.48	176.61	0
	Mean	8.04	13.59	865.55	3.29	60.95	486.01	98.71	5.50	37.77	33.80	103.34	77.34	251.10	1.85
	SE	0.02	0.41	22.49	0.32	18.11	16.83	8.07	0.16	1.69	0.93	4.36	2.40	5.53	0.28
High salinity water	*n*	5	-	-	-	-	6	6	6	6	6	6	6	6	6
	Max	6.72	-	-	-	-	381,960.22	61,385.00	16,739.00	5515.50	82,722.00	268,680.00	5698.00	824.44	44.90
	Min	6.55	-	-	-	-	294,484.30	12,587.50	2969.00	1052.00	34,250.00	191,460.00	0.00	218.00	0.00
	Mean	6.63	-	-	-	-	316,112.74	47,778.58	9123.33	2390.58	45,448.33	207,419.17	3686.50	458.91	28.62
	SE	0.03	-	-	-	-	12,452.19	6573.28	2401.33	625.92	6878.09	11,263.49	736.91	94.17	5.88
Surface water	*n*	7	7	7	7	7	7	7	7	7	7	7	7	7	7
	Max	8.49	26.10	696.00	4.63	249.00	377.11	58.00	4.08	46.60	30.60	117.00	72.60	198.00	4.21
	Min	7.78	10.70	360.00	2.36	132.00	192.81	21.40	0.00	30.99	13.27	22.40	25.66	105.82	1.82
	Mean	8.18	19.57	525.57	3.51	186.29	294.17	39.78	2.62	38.57	21.50	62.53	49.02	154.40	3.39
	SE	0.08	2.06	50.34	0.31	15.07	24.58	5.05	0.50	1.90	2.15	10.89	6.40	14.47	0.28

^a^ Number of hydrochemical sample, ^b^ Standard deviation.

**Table 2:** Principle component loadings for hydrochemical characteristics.

Parameter	PC1	PC2	PC3	PC4
pH	−0.75	0.51	0.26	−0.02
T	−0.17	−0.12	0.39	0.85
EC	0.99	0.06	0.06	0.01
DO	−0.1	−0.22	0.84	−0.07
ORP	0.07	−0.58	0.41	−0.49
TDS	0.99	0.06	0.06	0.01
Na^+^	0.94	−0.28	−0.14	0.09
K^+^	0.85	0.19	0.01	0.05
Ca^2+^	0.84	0.45	0.25	−0.07
Mg^2+^	0.93	0.26	0.18	−0.05
Cl^−^	0.99	0.09	0.08	0
SO_4_^2−^	0.89	−0.33	−0.16	0.11
HCO_3_^−^	0.64	0.6	0.09	−0.09
NO_3_^−^	0.83	−0.51	−0.04	0.09

**Table 3:** Alpha diversity indices of the microbial community.

Sample Types	Estimators	Sobs	Ace	Shannon	Simpson	Pielou_e
Phreatic water	Mean	974.83	1197	3.88	0.081	0.593
	SE	208.93	249.97	0.26	0.01	0.03
Artesian water	Mean	756.84	958.57	3.64	0.096	0.596
	SE	118.67	144.91	0.15	0.01	0.02
High-salinity water	Mean	811.75	1028.5	3.52	0.108	0.536
	SE	111.73	188.52	0.34	0.03	0.06
Surface water	Mean	3757.6	4666	5.20	0.058	0.639
	SE	719.98	735.71	0.65	0.02	0.06

## Data Availability

The data presented in this study are available on request from the corresponding author due to their complexity and ongoing use in further analyses.

## Supplementary Materials

The following supporting information can be downloaded at: https://www.mdpi.com/article/10.3390/life15081301/s1, Figure S1: Box plots present disparities in hydrochemical characteristics across distinct sample types based on Kruskal-Wallis Tests; Figure S2: PCA biplot of hydrochemical data excludes high-salinity groundwater; Figure S3: Rank-Abundance curves; Figure S4: Alpha-diversity multi-group Kruskal-Wallis test violin plot. Sobs (a), Ace (b), Shannon (c), Simpsom (d), Pielou_e (e). Table S1: The information of sampling sites; Table S2: Total variance interpretation of PCA; Table S3: Microbial community structure composition at Class level; Table S4: Microbial community structure composition at Family level; Table S5: Result of hierarchical partitioning; Table S6: Topological parameter of microbial co-occurrence network; Table S7: Result of βNTI.
