# Supplementary material for: Microbial Community Assembly Mechanisms of Groundwater Under Salinity–Oxygen Stress in the Golmud River Watershed, Northwest China
Source: Life (Basel). 2025 Aug 15;15(8):1301. doi: 10.3390/life15081301 (PMC12387591; doi:10.3390/life15081301)
Supplement: Supplementary file 1 [file life-15-01301-s001.zip › Supplementary_information.pdf]

SUPPLEMENTARY INFORMATION

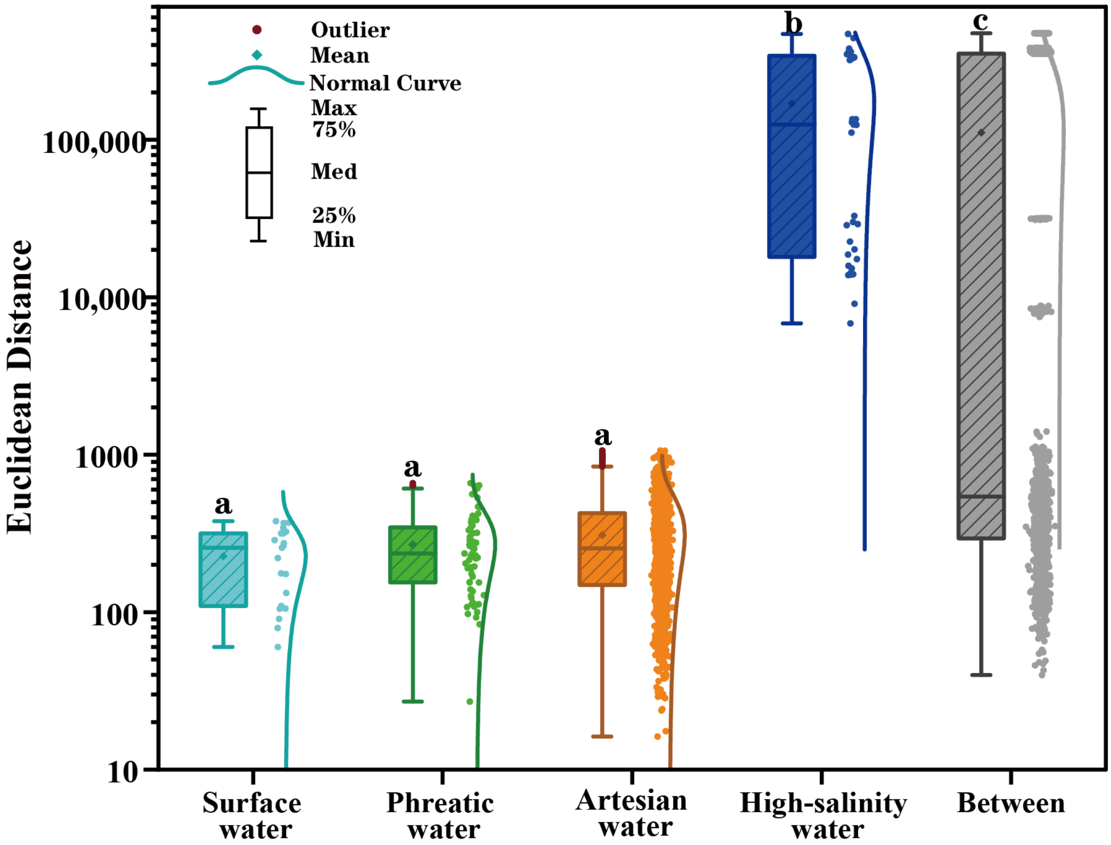

Figure S1. Box plots present disparities in hydrochemical characteristics across distinct sample types based on Kruskal–Wallis tests.

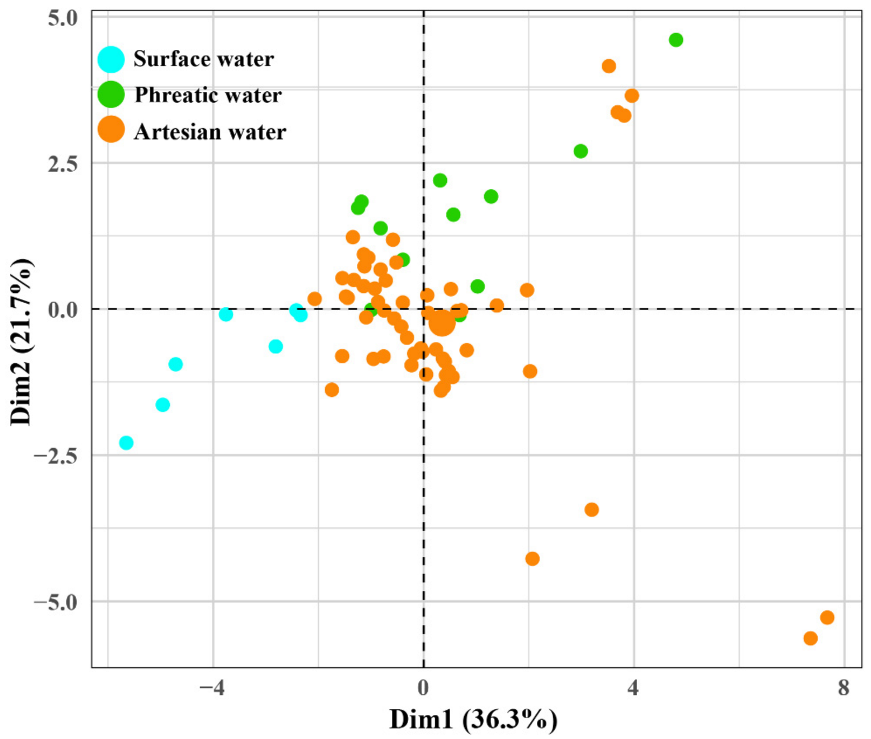

Figure S2. PCA biplot of hydrochemical data excludes high-salinity groundwater.

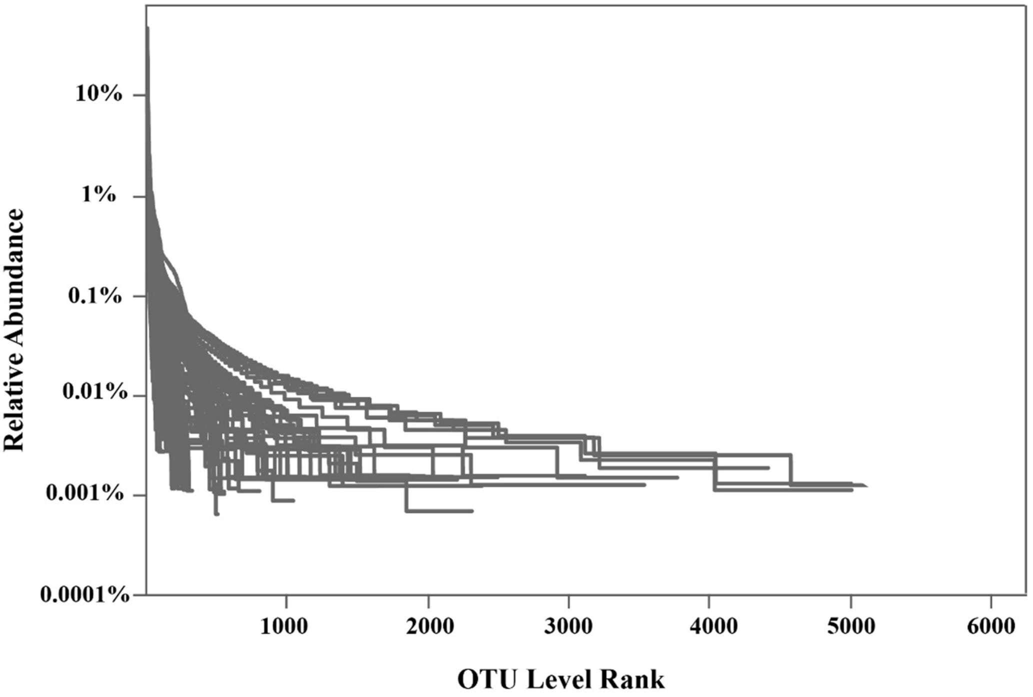

Figure S3. Rank–Abundance curves.

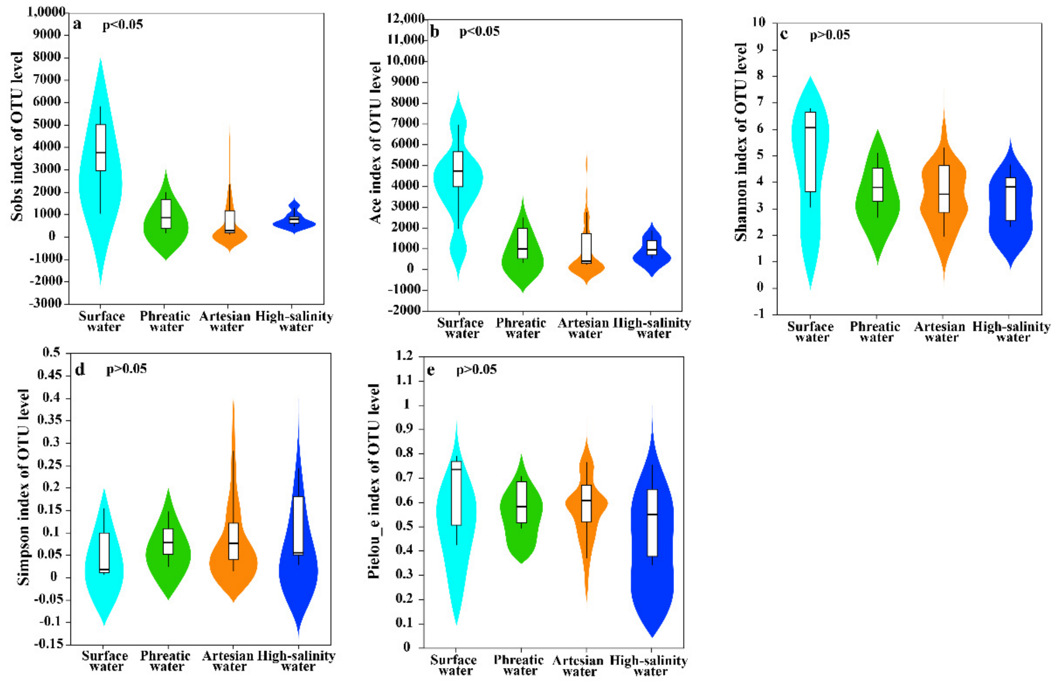

Figure S4. Alpha-diversity multi-group Kruskal–Wallis test violin plot.

Sobs (a), Ace (b), Shannon (c), Simpsom (d), Pielou_e (e).
